# Supplementary material for: MetaRibo-Seq measures translation in microbiomes
Source: Nat Commun. 2020 Jun 29;11:3268. doi: 10.1038/s41467-020-17081-z (PMC7324362; doi:10.1038/s41467-020-17081-z)
Supplement: Supplementary file 10 — Supplementary Data 7 [file 41467_2020_17081_MOESM10_ESM.zip › File2/Confidence_VeryHigh_Taxonomy/294481_out.krona.html]

Javascript must be enabled to view this page.

members
magnitude
magnitudeUnassigned
count
unassigned
taxon
rank

294481\_out

11

7
superkingdom
2

7
1239
phylum

7
186801
class

7
186802
order

7
186803
family

genus
265975
1


SRS077384\_contig\_number\_10596
1
species
652706

1213720
genus
6

6

SRS016456\_contig\_number\_2027SRS021528\_contig\_number\_32443SRS023617\_contig\_number\_16128SRS103971\_contig\_number\_34504SRS143423\_contig\_number\_12169SRS149078\_contig\_number\_contig-100\_630.134136
796942
species

4

SRS097912\_contig\_number\_6834SRS1055023\_contig\_number\_22852SRS142527\_contig\_number\_contig-100\_1214.217783SRS893352\_contig\_number\_contig-100\_8386.74293
